# A Computational Study of the Glycine-Rich Loop of Mitochondrial Processing Peptidase

**DOI:** 10.1371/journal.pone.0074518

**Published:** 2013-09-13

**Authors:** Tomáš Kučera, Michal Otyepka, Anna Matušková, Abdul Samad, Eva Kutejová, Jiří Janata

**Affiliations:** 1 Institute of Microbiology, Academy of Sciences of the Czech Republic, Prague, Czech Republic; 2 Department of Biochemistry, Faculty of Science, Charles University, Prague, Czech Republic; 3 Regional Centre of Advanced Technologies and Materials, Department of Physical Chemistry, Faculty of Science, Palacký University, Olomouc, Czech Republic; 4 Institute of Molecular Biology, Slovak Academy of Sciences, Bratislava, Slovakia; German Research School for Simulation Science, Germany

## Abstract

An all atomic, non-restrained molecular dynamics (MD) simulation in explicit water was used to study in detail the structural features of the highly conserved glycine-rich loop (GRL) of the α-subunit of the yeast mitochondrial processing peptidase (MPP) and its importance for the tertiary and quaternary conformation of MPP. Wild-type and GRL-deleted MPP structures were studied using non-restrained MD simulations, both in the presence and the absence of a substrate in the peptidase active site. Targeted MD simulations were employed to study the mechanism of substrate translocation from the GRL to the active site. We demonstrate that the natural conformational flexibility of the GRL is crucial for the substrate translocation process from outside the enzyme towards the MPP active site. We show that the α-helical conformation of the substrate is important not only during its initial interaction with MPP (i.e. substrate recognition), but also later, at least during the first third of the substrate translocation trajectory. Further, we show that the substrate remains in contact with the GRL during the whole first half of the translocation trajectory and that hydrophobic interactions play a major role. Finally, we conclude that the GRL acts as a precisely balanced structural element, holding the MPP subunits in a partially closed conformation regardless the presence or absence of a substrate in the active site.

## Introduction

The majority of mitochondrial proteins are synthesized as precursor proteins on cytosolic ribosomes and posttranslationally transported into the mitochondria. This process is facilitated by specific matrix-targeting signal presequences which are normally part of the N-termini of these proteins prior to their transportation. These preproteins are unfolded and imported into the mitochondrial matrix across a double membrane through protein translocation machinery comprising translocases of the outer [1] and inner mitochondrial membrane [2]. After precursor translocation, the targeting signal is no longer necessary and is proteolytically removed. Although several mitochondrial peptidases participate in preprotein processing, the most important role seems to belong to the mitochondrial processing peptidase (MPP) [3], since its deletion is lethal. Indeed, no inherited disorders have been linked with any mutants of MPP, indicating that its biological function is so vital that even relatively moderate disruptions to its activity are also likely to produce non-viable organisms. This may be linked to its essential role in mitochondrial biogenesis.

The architecture of MPP has been described based on the crystal structure of yeast MPP [4]. MPP consists of two structurally similar but non-identical subunits, α and β, which are encoded by two separate genes. Each subunit is comprised of two domains of 210 amino acid residues each which share nearly identical topology. A conserved HxxEH zinc-binding motif, which appears only in the catalytic β-MPP subunit, is located inside the enzyme cavity created by both subunits. Crystal structures of two different synthetic substrate peptides co-crystallized with mutant MPP showed the peptide bound in an extended conformation at the active site, forming a short series of β-sheet-like interactions with the β-sheets of β-MPP before proteolysis [5]. A common motif of almost all N-terminal signal presequences processed by MPP is an arginine residue located at position -2 with respect to the cleavage site (the R-2 motif) and additional arginine residues are usually located upstream in signal presequences. In the crystal structure, the R-2 residue was found to be interacting with E160 and D164, two conserved residues in the β-subunit which are close to the zinc-binding site (the R-2-binding motif). While the R-2 motif may be sufficient to indicate the cleavage site, it cannot be sufficient by itself for the initial substrate recognition, since MPP is able to specifically recognize a large variety of diverse mitochondrial signal presequences which generally have only low sequence similarity and vary in length.

Although it is known that both subunits are essential for MPP function [6-8], the mechanism of initial substrate recognition and the roles of each subunit in this process are still a matter of debate. Most studies have emphasized the importance of the α-subunit [9-12] in these processes and the ability of the signal presequences to form unstable α-helical amphipathic structures in hydrophobic environments, which seems to be important for the initial recognition of the presequence by MPP [13-15]. The most conserved part of all known α-MPPs is the glycine-rich loop (GRL; residues G^285^GGSFSAGGPGKGMYS^300^ in the yeast α-MPP), which has been shown to be essential for substrate binding [16]. The GRL also seems to be the structural element where the initial interaction between MPP and the signal presequence occurs [17]. The most important role in this process may be a hydrophobic interaction between two residues on one side of the presequence α-helix (often in positions -4 and +1, with respect to the cleavage site) with M298 and Y303 of the GRL. Since the GRL is situated at the entrance to the cleft formed by the MPP subunits, it is exposed to both the zinc-binding site and the substrate as it enters from outside the enzyme. Fluorescence resonance energy transfer experiments have shown that the C-terminus of the presequence interacts with the GRL following cleavage and points out of the enzyme cavity [18].

**Figure 1:**
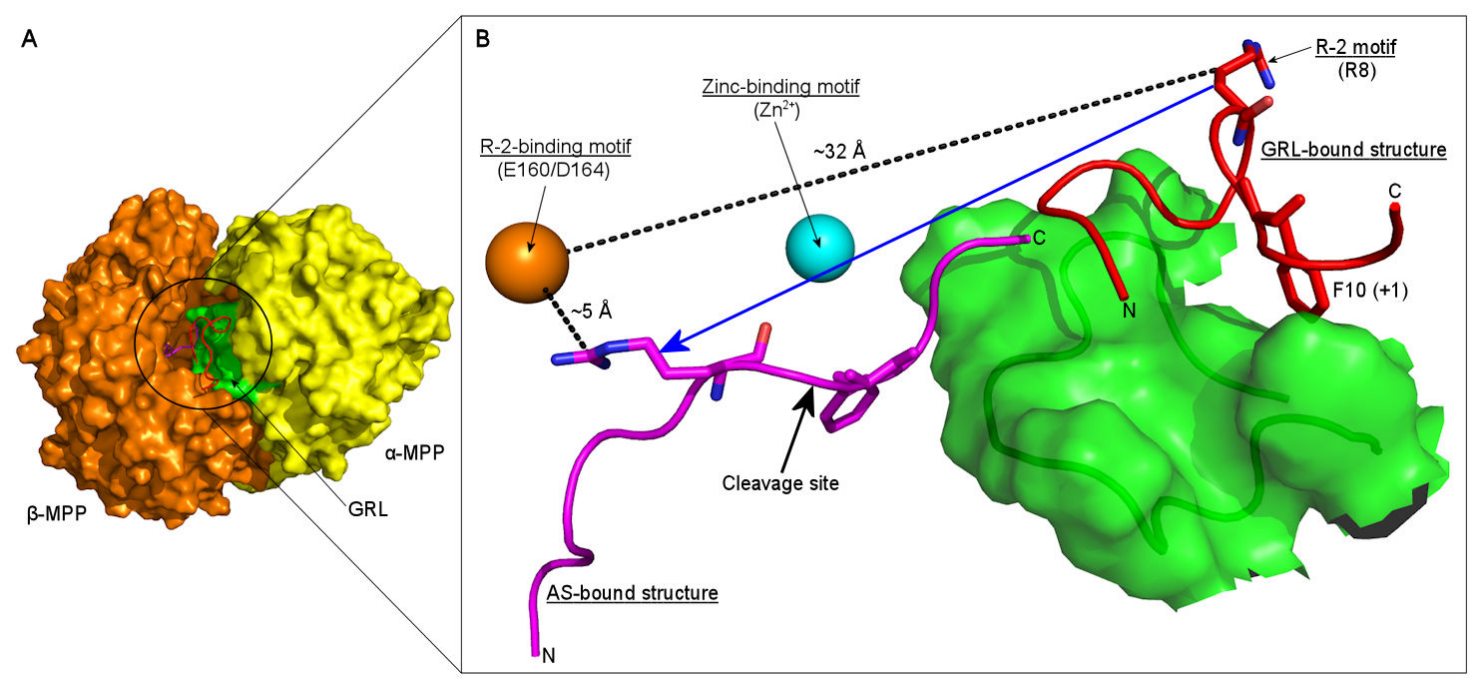
Scheme of substrate translocation from the GRL to the MPP active site. Panel A shows the overall structure of MPP. The van der Waals surface of α-MPP and β-MPP subunits and GRL is in yellow, orange and green, respectively. Panel B shows the conformation and position of the substrate during its recognition by the α-MPP GRL (*GRL-bound* *structure*; red tube) and just prior to its subsequent proteolysis in MPP active site (*AS-bound* *structure*; pink tube). The direction of substrate translocation between these two boundary positions is indicated by blue arrow. In the *GRL-bound* *structure* residue F10 contributes to the hydrophobic interaction with GRL and residue R8 (i.e. the R-2 motif) is exposed to the β-MPP subunit. In the *AS-bound* *structure* the substrate is bound in an extended conformation and its R8 residue interacts with the R-2-binding motif. The zinc-binding motif and R-2-binding motif are shown schematically as cyan and orange spheres which correspond to the zinc-ion and residues E160 and D164 residues of the β-MPP subunit. The distances between the substrate R8 residue and the R-2-binding motif in the *GRL-bound* and *AS-bound* *structure* are shown as dashed black lines.

In summary, it seems likely that the ability of the presequence to adopt context-dependent conformations during different steps of MPP’s action is a basic requirement for substrate recognition and processing. Although the MPP crystal structure was published more than a decade ago [4], very few studies have addressed the detailed operation of MPP. To date, the substrate binding and cleavage mechanism in the MPP active site (AS) has been described in detail [19] (*AS-bound structure* in Figure 1) and a mechanism of substrate recognition by the GRL has been outlined [17] (*GRL-bound structure* in Figure 1), however the mechanism of substrate translocation from the GRL to the MPP active site has not yet been investigated. Since the whole process is complex and involves interactions of larger regions rather than single amino acids, the experimental approach using amino acid point mutations is problematic. Thus, the aim of the present study is to determine the precise role of the GRL in the different steps of substrate processing, using non-restrained and targeted molecular dynamics simulations, which may provide new insights into MPP substrate recognition and processing mechanisms which are difficult to acquire experimentally.

## Results

### Substrate translocation from GRL to MPP active site

Targeted molecular dynamics (TMD) simulation was carried out to examine the translocation of the substrate from the GRL (i.e. from the place of the initial recognition of the signal presequence) to the MPP active site (i.e. to the place where the signal presequence cleavage occurs). Both boundary positions are displayed in Figure 1, as *GRL-bound* and *AS-bound structures*, respectively. A peptide derived from the malate dehydrogenase (MDH: residues L^2^SRVAKRAFSST^13^; the R-2 motif is underlined) signal presequence was chosen as a model substrate.

**Figure 2:**
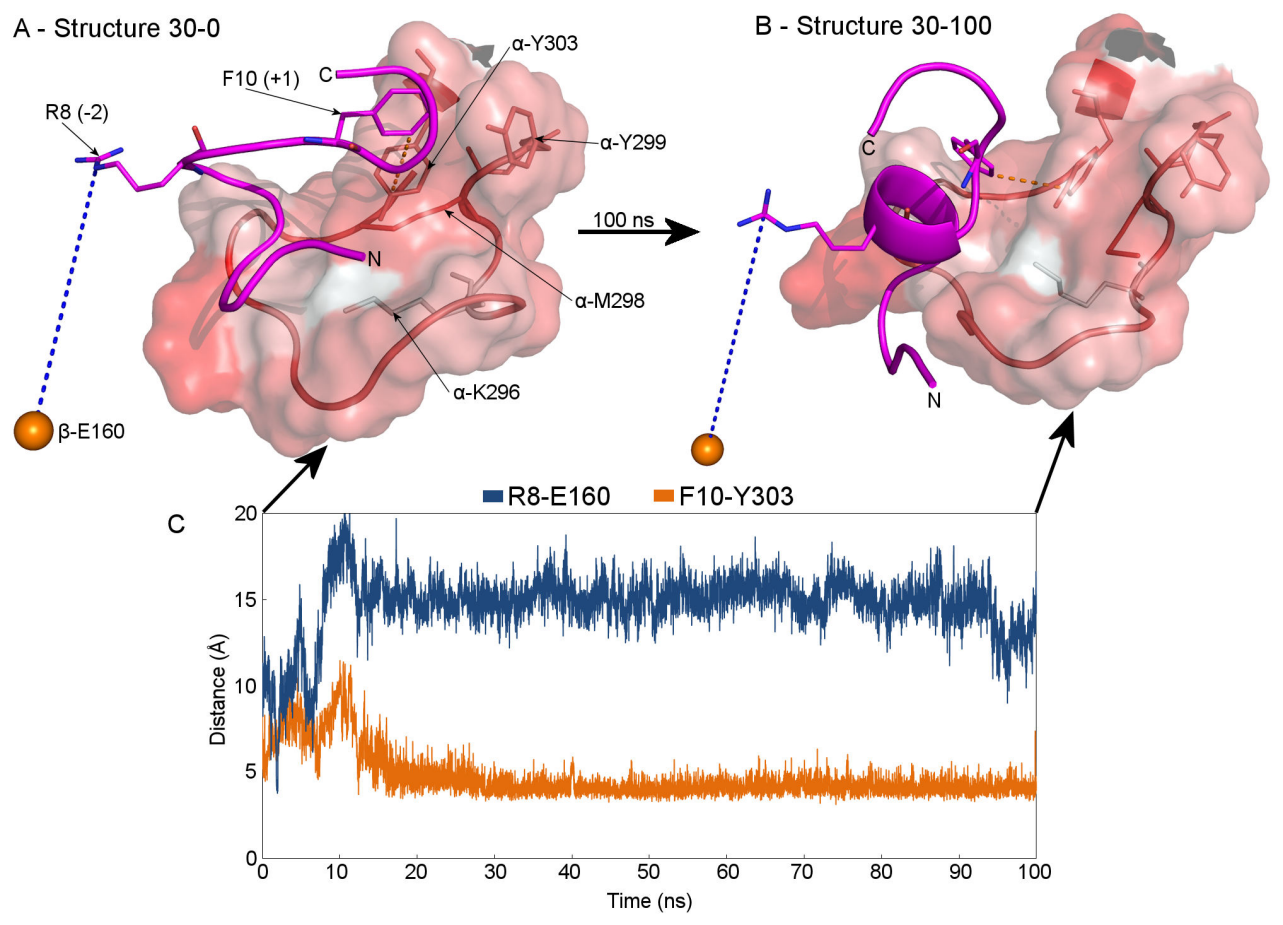
GRL-substrate interaction after the substrate has completed the first third of its translocation. Panel A (Structure 30-0) shows a snapshot from the targeted MD simulation corresponding to the situation after 0.48 ns out of a 1.6-ns-long restraint period. Panel B (Structure 30-100) shows the GRL-substrate interaction after a 100-ns-long, non-restrained MD simulation performed on *Structure 30-0*. The GRL lies at the entrance to the active site cavity between the α-MPP and β-MPP subunits and is displayed as a semi-transparent van der Waals surface, colored according to residue hydrophobicity [34]. The backbone trace can be seen within this surface and is displayed as a tube. The backbone trace of the substrate is displayed as a magenta tube. Residues K296, M298, Y299 and Y303 of the GRL and residues F10 and R8 (i.e. the R-2 motif) of the substrate are shown as sticks. The α and β prefixes in residue names refer to the α- or β-MPP subunits, respectively. The numbers in brackets show the position of the given residue with respect to the substrate cleavage site. The orange sphere shows the positions of the δ-carbon of the E160 residue and thus represents schematically the R-2-binding motif. The distance between the ζ-carbon atom of the R8 residue and the R-2-binding motif (i.e. δ-carbon of the E160) and the distance between the δ-carbon of residue F10 and the hydrophobic patch of the GRL (represented by the ε-carbon of the α-subunit Y303 residue) are shown as dashed lines in blue (“R8-E160”) and orange (“F10-Y303”), respectively. Panel C shows these two distances over the course of the non-restrained MD simulation. Note that (i) the N-terminus of the substrate shifted while the substrate has curled into an α-helix. Moreover, note that during the whole non-restrained MD simulation (ii) the GRL-substrate interaction was stable (the R8-E160 and F10-Y303 distances were largely unchanged) and (iii) the substrate F10 residue interacted with the hydrophobic patch created by GRL residues M298 and Y303.

**Figure 3:**
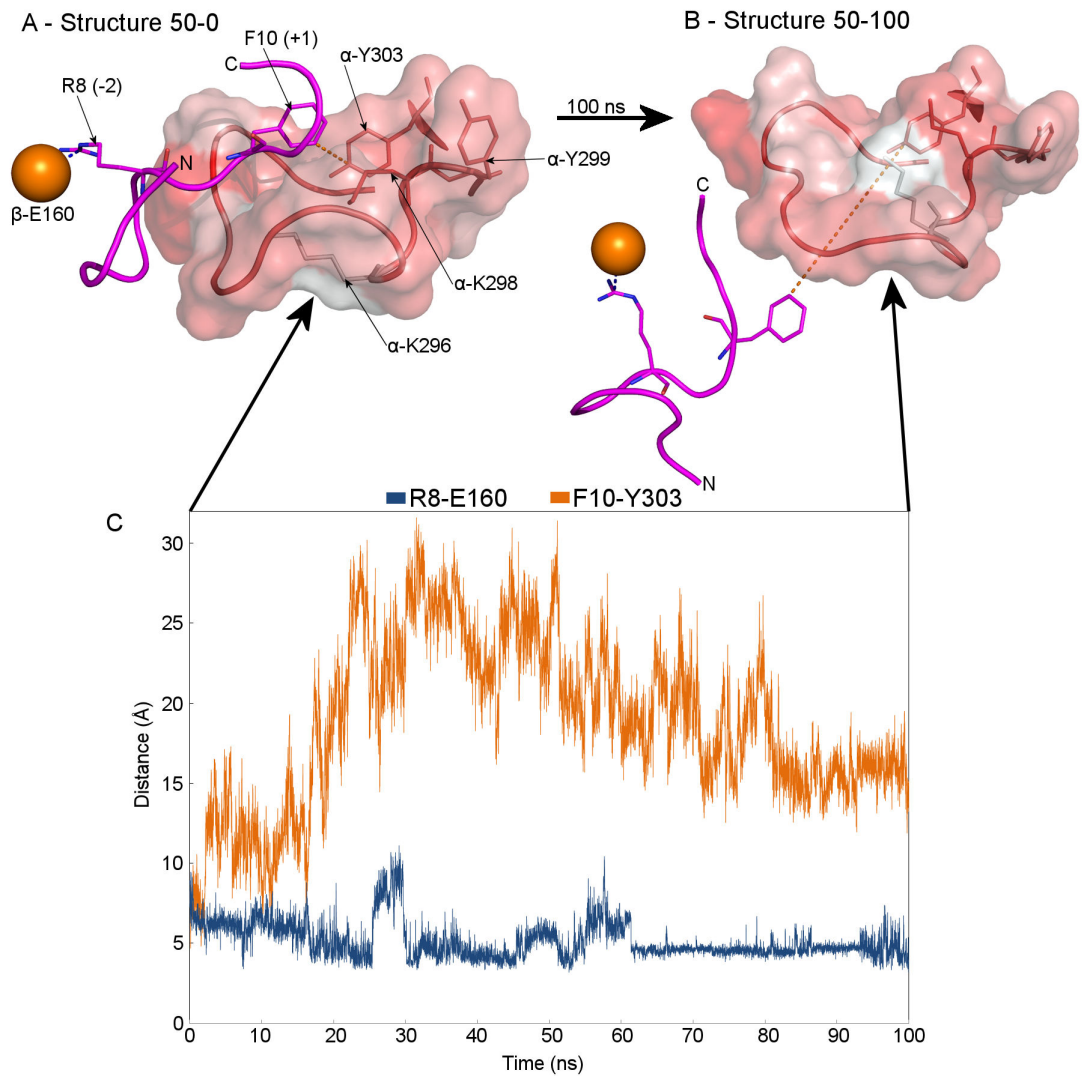
GRL-substrate interaction after half the substrate translocation. Panel A (Structure 50-0) shows a snapshot from the targeted MD simulation corresponding to the situation at time 0.84 ns of a 1.6-ns-long restraint period. Panel B (Structure 50-100) shows the GRL-substrate interaction after a 100-ns-long non-restrained MD simulation performed on *Structure 50-0*. The structural elements are represented as in Figure 2. Note that (i) the substrate has now shifted completely away from the GRL towards the MPP active site, (ii) the R8-E160 distance decreased from 8 to 4 Å and that (iii) the R8 residue has reoriented towards the R-2-binding motif.

We performed three TMD simulations with different restraint durations (Table 1) and since all of them provided the same results, for subsequent analysis we selected the longest one, with a restrain period of 1.6 ns and a total simulation period of 1.8 ns (Video S1). Two snapshots along the substrate translocation trajectory were chosen for further detailed analysis: one at 0.48 ns, representing a point one-third of the way through the translocation, and a second at 0.84 ns, corresponding to roughly the mid-point of the translocation (Structures 30-0 and 50-0 in Figures 2 and 3, respectively). These two structures representing the GRL-substrate interaction at the two selected snapshots were subsequently subjected to a 100-ns-long non-restrained MD simulation to study the GRL-substrate interaction in detail. The MPP dimer remained in its stable, partially-closed conformation during the whole TMD simulation, as documented by an analysis of root-mean-square deviation (RMSD) of Cα atoms (Figure S1). The second half of the translocation trajectory was not examined because the substrate was moving through free space, without supporting interactions with the surrounding MPP residues and therefore did not represent a reliable model of the translocation process at this stage.

**Table 1:** Targeted MD simulation parameters.

	**Starting RMSD**	**Restrain duration**	**Total duration**
**TMD1**	24.9 Å	0.5 ns	1.0 ns
**TMD2**	24.9 Å	1.0 ns	1.2 ns
**TMD3**	24.9 Å	1.6 ns	1.8 ns

***Structure30-0*** in Figure 2 represents the situation after the substrate has completed the first third of its movement (0.48 ns from a 1.6-ns-long restrain period, i.e. ~30%). Two distances were used to monitor the position of the substrate with respect to the GRL of the α-MPP subunit and the active site of the β-MPP subunit during the subsequent non-restrained MD simulation: (i) the distance between the R-2 motif of the substrate (represented by the ζ-carbon atom of the R8 residue) and the R-2-binding motif of β-MPP (represented by the δ-carbon of the β-subunit E160 residue), and (ii) the distance between the hydrophobic F10 residue in position +1 relative to the substrate cleavage site (represented by its δ-carbon) and the hydrophobic patch of the GRL (represented by the ε-carbon of the α-subunit Y303 residue). These data suggest that the substrate continued to interact with GRL without significant changes in its position and distance from the enzyme active site. In contrast, examining the size of the interaction surface between the substrate and the GRL reveals that the interaction surface decreased from ≈250 Å^2^ at the beginning of the simulation to ≈180 Å^2^ at the end (a decrease of 30%). At the beginning of the non-restrained MD simulation, the substrate had no defined secondary structure. Following non-restrained MD simulation, the substrate acquired a stable α-helical conformation and, although it still interacted with the GRL, the N-terminus was reoriented towards the enzyme active site (***Structure30-100*** in Figure 2). Although the interaction surface decreased by 30% at this stage the substrate F10 residue still interacted with a hydrophobic patch created by M298 and Y303 of the GRL.

***Structure50-0*** in Figure 3 represents the situation when the substrate has reached the mid part of its trajectory (0.84 ns of 1.6 ns total, i.e. ~50%). Here, at the beginning of the non-restrained MD simulation the substrate remained in an undefined, partially extended conformation, but still in contact with the GRL during the first 15 ns of the simulation. Interestingly, later during the simulation, the whole substrate distinctly shifted towards the enzyme active site (***Structure50-100***). The shift of the substrate is documented by a gradual increase in the F10-Y303 distance during the first half and a subsequent slight decrease during the second part of the simulation period and by a decrease in the interaction surface between the substrate and GRL from ≈120 Å^2^ at the beginning to 0 Å^2^ at the end of the non-restrained MD simulation. The substrate R8 residue (i.e. the R-2 motif) was reoriented towards the β-subunit E160 residue, the part of the R-2-binding motif that participates in substrate binding in the MPP active site.

### Structural role of GRL for the MPP dimer stability

An all-atomic, non-restrained MD simulation with explicit solvent was carried out to gain insights into the structural dynamics of MPP. The structure of yeast wild-type MPP (WT MPP) with bound substrate in its active site was taken from the Protein Data Bank. The substrate is derived from the CytC oxidase IV (COX IV: residues S^7^IRFFKPATRT^17^; the R-2 motif is underlined) signal presequence. The unbound structure of WT MPP was produced by removing the substrate from peptidase active site. Analogously, two models of mutant MPP with deletion of the GRL (ΔGRL MPP) were constructed - a ΔGRL MPP with the substrate bound in its active site and a ΔGRL MPP with no bound substrate.

**Figure 4:**
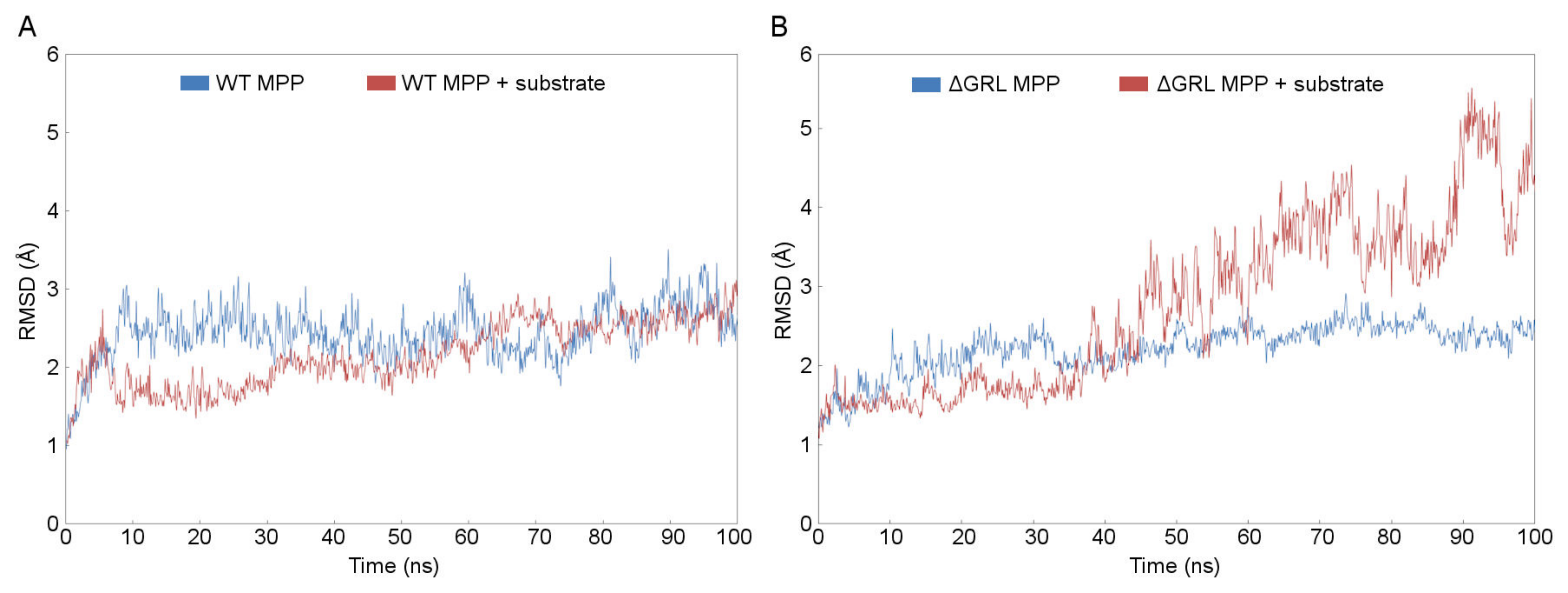
Time-based RMSD of backbone Cα atoms of WT MPP and ΔGRL MPP. Panel A shows the change in the RMSD of the WT MPP structure over the course of a 100 ns simulation with respect to the initial model. Runs both with (red) and without (blue) a active site-bound peptide substrate are shown. Panel B shows the same information, but for the ΔGRL MPP structure. The ΔGRL MPP structure was produced by deleting residues 285-300 from the α-MPP structure. Figure S2 displays the same information in 2D RMSD plots.

The analyses of RMSDs of MPP backbone Cα atoms were chosen as a tool for monitoring the trajectory stability and conformational changes (Figure 4). The 2D representations of RMSD are shown in Figure S2. The systems representing WT MPP with and without a bound substrate showed stable conformations during the entire production phase of the simulations (Figure 4-A). After 10 ns, the RMSD of WT MPP fluctuated slightly around a mean value of 2.5 Å (±0.5 Å). Binding of the substrate to the MPP active site seemed to stabilize the MPP conformation, however, since the overall backbone RMSD decreased by 0.7 Å during the first 30 ns of simulation and gradually rose to a mean value of 2.5 Å at the end of the simulation. Examining the size of the interaction surface between the two MPP subunits reveals that it increased from ≈2100 Å^2^ at the beginning of the simulation to ≈2300 Å^2^ at the end (Table 2). In contrast, the unbound structure showed the opposite trend, with the interaction surface decreasing from ≈2100 Å^2^ at the beginning to ≈1800 Å^2^ at the end. (It is important to note that these numbers include only the contact area between the two subunits and have no contribution by the substrate.) These data suggest that binding of the substrate in the WT MPP active site strengthens the dimer interface.

**Table 2:** Interaction surfaces between the α and β-subunits of MPP and between the β-subunit and the bound peptide substrate in MPP active site.

	**α-MPP versus β-MPP**		**Substrate versus β-MPP**	
	**Start value**	**End value**	**Start value**	**End value**
WT	2110 Å^2^	1812 Å^2^	-	-
WT + substrate	2110 Å^2^	2336 Å^2^	886 Å^2^	886 Å^2^
ΔGRL MPP	1850 Å^2^	1907 Å^2^	-	-
ΔGRL MPP + substrate	1850 Å^2^	1428 Å^2^	886 Å^2^	861 Å^2^

In the absence of the substrate, the ΔGRL MPP enzyme structure did not undergo significant conformational changes. This is illustrated by the fact that its backbone RMSDs fluctuated around a mean value of 2.5 Å for the second half of the MD simulation (Figure 4-B). These fluctuations are smaller than for the WT MPP since the loss of large flexible GRL reduced the overall variability. On the contrary, binding of the substrate appeared to destabilize the ΔGRL MPP dimer. During the MD simulation, the RMSD didn’t reach a plateau and instead was continuously increasing, with a RMSD value of 4.5 Å at the end of simulation, almost double that observed in the WT MPP simulation. There were also wide fluctuations of about 1 Å. Examining the amount of surface area per monomer buried on dimerization shows that it increased slightly from ≈1850 Å^2^ to ≈1900 Å^2^ for the model lacking bound substrate and decreased substantially from ≈1850 Å^2^ to ≈1400 Å^2^ for the model with bound substrate (Table 2).

During both WT MPP and ΔGRL MPP simulations, the substrate remained bound in the MPP active site, as shown by an assessment of the interaction surface area between the substrate and the β-MPP subunit (Table 2). In addition, RMSD of Cα atoms of the substrate was calculated between the structures at the beginning and the end of MD simulations. RMSD of the full-length substrate was 2.5 Å and, naturally, the most flexible parts were the C- and N-terminus of the substrate. When one or two residues were removed from each termini of the substrate, the RMSD decreased and reached 1.2 Å and 0.5 Å, respectively.

**Figure 5:**
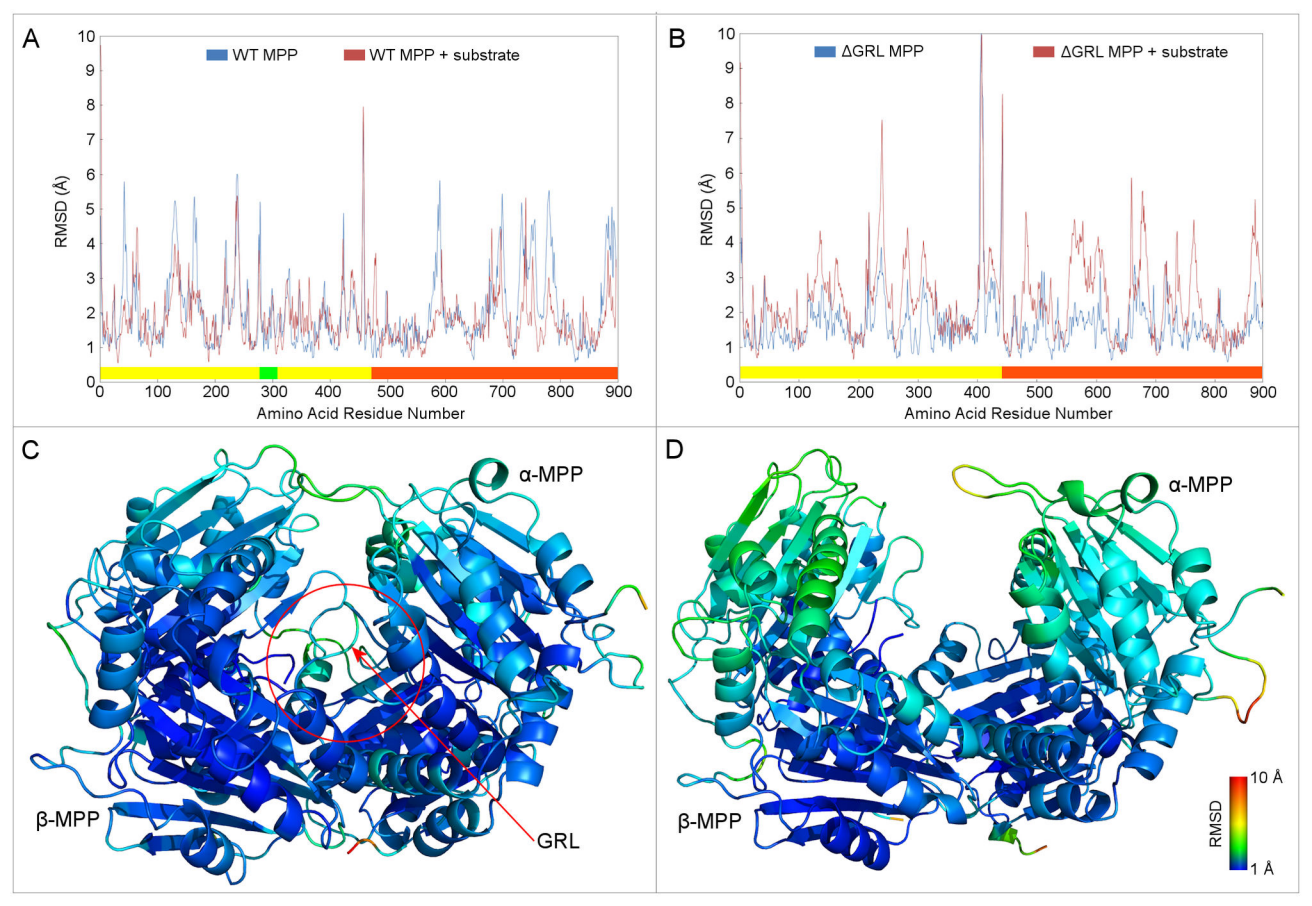
Residue-based RMSD of backbone Cα atoms of WT MPP and ΔGRL MPP. Panels A and B show the RMSD per residue of the WT MPP and ΔGRL MPP structures, respectively. The red lines show the RMSD of the structures in the presence of the active site-bound substrate and the blue lines represent the unbound structures at the end of a 100-ns-long MD simulation. The bars along the *x*-axes indicate the residues which belong to the α (yellow) and β-MPP (orange) subunits, while the green bar in panel A indicates the position of the GRL (residues 285-300) which was deleted in the ΔGRL MPP structure. Panels C and D show, respectively, the WT MPP and ΔGRL MPP structures colored to reflect their per-residue RMSDs and structurally aligned according to the parts with the lovest fluctuation of RMSD. Both structures are colored according to RMSD scale bar in the bottom right corner of panel D. The red circle marks the position of the GRL. Note that the ΔGRL MPP dimer appears to be more open than the WT structure and that the RMSDs of the areas farthest from the dimer interface are notably higher.

To more directly compare WT MPP and ΔGRL MPP, a residue-based RMSD analysis was used which indicated that the differences in backbone flexibility arise from the presence of the substrate in the enzyme site (Figure 5). For WT MPP, the presence of the substrate did not affect the enzyme structure significantly, though a small decrease in the β-MPP RMSD might have been present (Figure 5-A). On the other hand, for ΔGRL MPP, the presence of substrate in the enzyme cavity caused a large increase in the RMSD, indicating that the enzyme structure was significantly affected (Figure 5-B). In general, the most flexible parts of both MPP structures are their surface loops and N- and C-termini. The deletion of the GRL destabilized those parts of the MPP subunits that are not in direct contact and farthest from the dimer interface (Figure 5-C and D). Thus, the ΔGRL MPP dimer with bound substrate appears to be more open and less stable than the WT structure.

A radius of gyration analysis supports this conclusion (Figure S3). According to this analysis, when the substrate binds to the active site of WT MPP, the radius shrank, indicating that the enzyme dimer had become more compact. In contrast, the structure of ΔGRL MPP bound to a peptide substrate had a larger radius of gyration which increased during the course of the simulation. This indicates that the dimer became looser or more open and less stable.

## Discussion

Our previous results, obtained using tryptophan fluorescence measurements and MD simulations, showed that the GRL of α-MPP is the site where the primary interaction between the signal presequence (as a substrate) and MPP occurs, and suggested that the substrate’s α-helical conformation was important for this interaction [16]. In this study we employed MD simulations to study the role of the GRL in the process of substrate translocation from the GRL to the MPP active site and also its role in the tertiary and quaternary structures of MPP.

### GRL as an active element during substrate translocation

The large number of glycine residues, together with the weak electron density for the GRL in the MPP crystal structure, indicates that this loop is highly flexible. Using a targeted MD simulation, we simulated the process of substrate translocation from the GRL to the MPP active site (Figure 1). During this process, the GRL undergoes significant conformational changes in the part containing residues 289-293 in α-MPP (Video S1). However, these changes did not appear to affect the MPP subunit interaction, since RMSD fluctuated around an average value of 1.5 Å (Figure S1) and interaction surface between the two MPP subunits fluctuated between values 2150 and 2250 Å^2^ (Table S1) during the whole simulation. We chose two steps along the trajectory and studied the enzyme-substrate interaction by non-restrained MD simulation.

First we studied the enzyme-substrate interaction when the substrate had finished the first third of its trajectory (Figure 2). To our surprise, the substrate, which at this stage had no definable secondary structure, acquired at the end of the following non-restrained MD simulation an α-helical loop stabilized by a hydrophobic interaction between hydrophobic residues on one side with a hydrophobic patch of the GRL and by hydrogen bonds between positively charged arginine residues on the other side of the α-helix with negatively charged residues on the β-MPP subunit. Furthermore, the process of α-helix loop folding was accompanied by reorientation of the N-terminus of the substrate towards the enzyme active site. Thus, although initially we hypothesized that hydrophobic interactions play an important role only during the initial substrate recognition [17], now it seems to be likely that hydrophobic interactions take significant part also later during the substrate translocation process.

**Figure 6:**
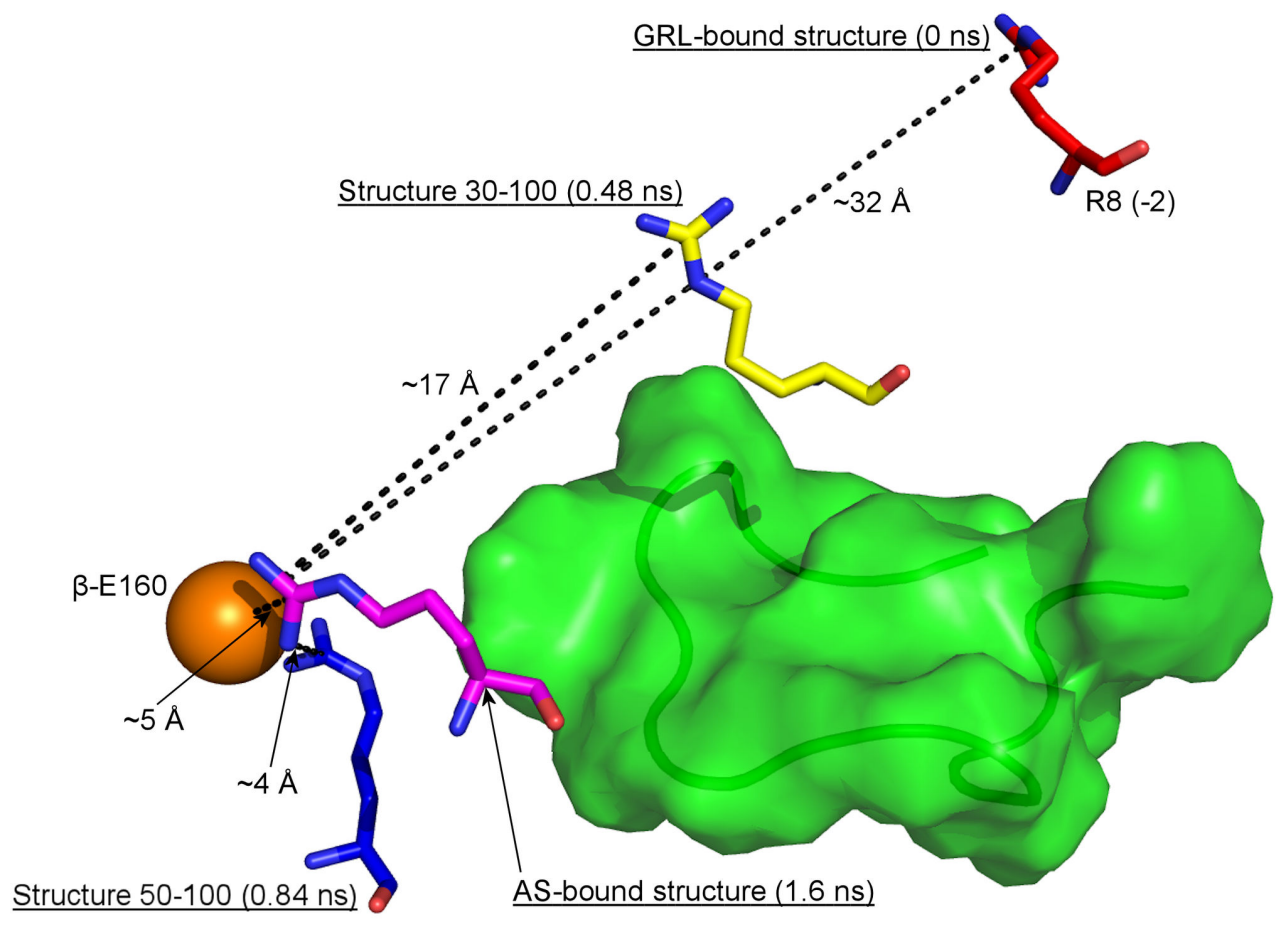
Scheme showing the positions of the R8 residue along the substrate translocation trajectory. The R8 residue is located in position -2 relative to the substrate cleavage site and thus represents the R-2 motif. The R-2-binding motif is shown schematically as an orange sphere representing the δ-carbon atom of the β-subunit E160 residue and the GRL is displayed as a green semi-transparent van der Waals surface whose backbone chain is displayed as a tube. In the *GRL-bound* *structure* the substrate is bound to GRL and residue R8 (red sticks) is exposed to the β-MPP subunit. In the *AS-bound* *structure* (purple sticks), on the other hand, the substrate is bound in the MPP active site and the R8 residue interacts with the R-2-binding motif. The positions of the R8 residues in *Structure 30-100* (yellow sticks) and *Structure 50-100* (blue stick) were obtained at the end of a 100-ns-long non-restrained MD simulation performed on the structures corresponding to the snapshots from a targeted MD simulation when the substrate reached the first third and the mid part of its translocation trajectory. The numbers in brackets mark the time steps along the substrate translocation trajectory. The distances between the R8 residue and the R-2-binding motif are shown as dashed black lines. Note that while *Structure 30-100* has the R8 residue oriented away from the GRL, in *Structure 50-100* it is oriented towards the R-2-binding motif and its distance from it is almost the same as in the *AS-bound* *structure*.

Subsequently, the substrate reached the mid part of its translocation trajectory (Figure 3). At this stage the substrate R8 residue (i.e. the R-2 motif) did not interact with either the E160 or D164 residues of β-MPP (i.e. the R-2-binding motif), as they did in the MPP crystal structure. However, during the following non-restrained MD simulation the substrate shifted notably deeper towards the MPP active site and the R-2 residue was reoriented in several steps towards the E160 residue of the R-2-binding motif. The position of R8 at different stages of substrate translocation is shown schematically in Figure 6.

The tendency of the substrate to shift spontaneously towards the MPP active site is illustrated by the lengthening of the distance between the substrate F10 and GRL Y303 residues and the simultaneous approach of the substrate R8 residue (i.e. the R-2 motif) to the R-2-binding motif of the β-MPP subunit (Figure 3-C). An interaction surface analysis between the substrate and the GRL further confirms this tendency, which becomes even more evident later after the substrate has reached the mid part of its translocation.

The role of the GRL has also been studied by site-directed mutagenesis. Nagao et al. reported that MPP with mutations F289A, F289L, K296A or M298A in the GRL of the α-MPP subunit had 10-fold less affinity for substrate peptides than did the wild-type and that their activities decreased to 1% [16]. However, the mutation M298L decreased the activity by only half, suggesting that the important feature of the amino acid residue in this position is its hydrophobic character. Mutations of the partially hydrophobic residue Y299 to serine and alanine had the same effect. The authors used a pull-down assay to investigate the effect of these mutations on the subunit interaction and suggested that the decrease in activity is not due to an incorrect or insufficient interaction between the MPP subunits. Our findings suggest, however, that the subunit interactions may be weakened as a result of these point mutations, though perhaps they remain strong enough to allow the MPP subunits to interact during the pull-down assay. Moreover, we conclude that K296 of the GRL appears to act as a stabilizing element for the whole GRL: In the absence of a substrate, it restricts the flexibility of the GRL and therefore controls the size and shape of the hydrophobic patch created by its side chain together with the M298, Y303 and Y299 residues.

### GRL keeps MPP dimer in a partially closed conformation

A MPP has been found in the mitochondria of different species, including yeast, mammals, plants and protozoa. Moreover, analogs of MPP have recently been found in hydrogenosomes [20], the organelles evolutionary-related to mitochondria. Specifically, a hydrogenosomal processing peptidase (HPP), a MPP-like protein, was characterized from the hydrogenosomes of the human parasite *Trichomonas vaginalis* [21]. MPP-like proteins are also present in bacteria [22] and the α-proteobacterial processing peptidase of *Rickettsia prowazekii* (RPP) is thought to be the closest modern example for the progenitor of MPP [23]. Several other bacterial peptidases have recently been described [24,25], including an example of the heterodimeric M16 peptidase from *Sphingomonas* sp. [26]. This one will be discussed in more detail below.

**Figure 7:**
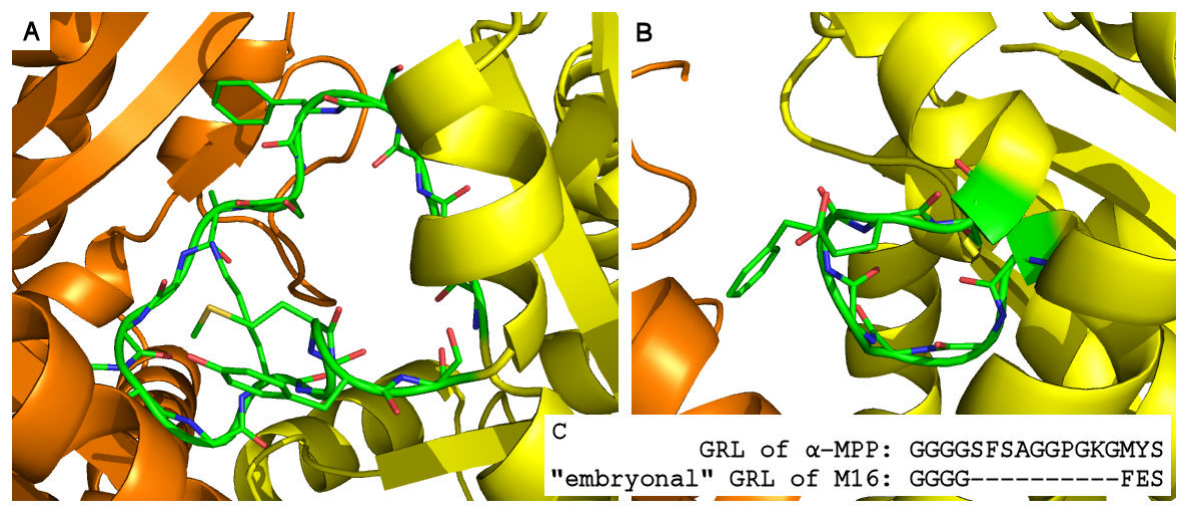
Detail structures of GRLs of MPP and M16 peptidase from *Sphingomonas sp.* Panel A shows the GRL region of MPP while panel B shows the “embryonal” GRL of M16 peptidase. The α-subunit is shown in yellow, β-subunit in orange, and the GRL in green. The side chains of amino acids 285–300 of the MPP GRL and 290–296 of the M16 peptidase GRL are represented as sticks. Panel C shows a sequence alignment of both GRLs.

A common trait of all these peptidases is the presence of a partial or complete GRL-like structural element, which may influence their quaternary structure and therefore their mechanism of action. Although the GRL-like element of HPP differs from the GRL of α-MPP in the sequence (Figure S4), the GRLs of all eukaryotic MPPs are highly conserved, indicating that they are vitally involved in the protein’s biological function. The bacterial peptidases, on the other hand, contain just “embryonal” GRL, if any. An example is shown in Figure 7.

The influence of the GRL on the MPP tertiary and quaternary structure was studied by non-restrained MD simulations performed on WT and ΔGRL MPP models, both with and without a peptide substrate bound in the active site. Analyses of RMSD, the interaction surface and the radius of gyration showed that while the WT MPP became more compact, ΔGRL MPP acquired a less compact and more open conformation.

Several experimental studies have been published which focus on the GRL. A pull-down assay of various α-MPP GRL deletion mutants by β-MPP-His_6_ revealed that these subunits continued to associate stoichiometrically, leading the authors to suggest that the GRL does not influence the association between subunits [16]. Furthermore, an MPP dimer containing an α-MPP with residues 249-287 deleted was still able to cleave the malate dehydrogenase (MDH) presequence, albeit with lower processing activity than that of WT MPP, but could process longer presequences only inefficiently [23]. On the other hand, the deletion of G292 makes α-MPP alone unable to bind (i.e. recognize) the short presequence of yeast MDH [17].

We are proposing a unifying interpretation of these contradictory experimental results. We suggest that GRL is a crucial structural element, which is responsible for holding the MPP binding cleft in a finely adjusted, partially closed conformation. Larger deletion of this part does not prevent the MPP subunits from associating, but in the presence of substrate it does cause this cleft to adopt a more open dimer conformation, which may alter the peptidase specificity, but preserve the partial peptidase activity. On the other hand, the deletion of only one amino acid may change the conformation of GRL itself and thus “close the entrance” to the MPP active site. The normal, partially closed conformation of WT MPP is not affected by the presence or absence of a substrate in its active site, unlike *Sphingomonas* sp. M16 peptidase, the peptidase that lacks a GRL, which adopts distinct closed and open conformations, depending on whether there is or is not a substrate bound to its active site [26].

## Models and Methods

### Models for the substrate translocation study

#### The GRL-bound structure

The *GRL-bound structure* of MPP in its initial interaction with a peptide derived from the malate dehydrogenase presequence (MDH: residues L^2^SRVAKRA↓FSST^13^; the arginine residue in position -2 relative to the cleavage site, i.e. the R-2 motif, is underlined) was built based on the MPP model described in Dvorakova-Hola et al. [17]. Here, the model of MPP with aldehyde dehydrogenase (ALDH) presequence peptide bound to GRL was constructed on the assumption that GRL may recognize its substrate in a similar way as the mitochondrial Tom20 receptor (part of translocase system of the outer mitochondrial membrane). The MPP structure with GRL-bound ALDH was generated by superposition with the analogous Tom20 receptor protein (PDB ID: 1OM2) and the model obtained was confirmed by tryptophan fluorescence experiments. In our study, the amino acid residues of the ALDH presequence were substituted by those of the MDH presequence and a final *GRL-bound structure* (Figure 1) was obtained at the end of a 100-ns-long non-restrained MD simulation performed on the initial model to relax all possible strains that may have arisen during model building.

#### The AS-bound structure

The *AS-bound structure* could not be built directly based on the crystal structure of MPP bound to MDH (PDB ID: 1HR9 with resolution 3.01 Å), since the structure of the GRL misses here. Instead, the initial *AS-bound structure* was built based on the crystal structure of MPP bound to a peptide derived from the COX IV presequence (PDB ID: 1HR8 with resolution 2.70 Å) where the amino acid residues of the COX IV presequence were substituted by those of the MDH presequence. In the next step, the presequence was extended by building an additional four residues, FSST, at the C-terminus. The final *AS-bound structure* was obtained from a 100-ns-long non-restrained MD simulation that was carried out on the initial model to relax all possible strains that may have arisen during model building (Figure 1).

### Models for the study of the structural role of GRL

Two MPP crystal structures deposited in Protein Data Bank were used for models preparation – the structure of MPP without and with bound peptide substrate in its active site (PDB ID: 1HR6 with resolution 2.50 Å and 1HR8 with resolution 2.70 Å, respectively). The structure of wild-type MPP (WT MPP) without bound substrate was taken directly from the 1HR6 structure. However, the model of WT MPP with bound substrate could not be generated from the 1HR6 structure, as the GRL segment (288-292) shows only weak electron density and is unresolved in the crystal structure. Thus, the WT MPP with bound substrate was constructed by combining the 1HR6 and 1HR8 structures – i.e. both structures were aligned and while the structure of the WT MPP was taken from the 1HR6, the structure of the bound presequence was taken from the 1HR8. The bound substrate was a synthetic peptide derived from the CytC oxidase IV signal presequence (COX IV) with residues S^7^IRFFKPATRT^17^↓ (the arginine residue in position -2 relative to the cleavage site, i.e. R-2 motif, is underlined). A model of mutant MPP with deletion of the GRL was constructed based on a sequence alignment of the WT MPP α-subunit and the *Rickettsia prowazekii* processing peptidase, a peptidase which lacks a large part of the GRL sequence (Figure S4). In addition, the previous experimental work was also kept in mind [27,28]. Thus, residues 285-300 of the α-MPP subunit were deleted, the N and C-termini of the resulting gap were linked and two models were prepared, a ΔGRL MPP with the COX IV peptide substrate bound in its active site and a ΔGRL MPP with no bound substrate.

### Targeted MD simulations

Targeted MD (TMD) simulations were employed to study the process of substrate translocation from the site of its initial recognition (i.e. GRL) to the MPP active site.

TMD simulations were performed in AMBER [29] with an additional term to the energy function based on the mass-weighted RMSD of a set of atoms in the *GRL-bound structure* compared to the *AS-bound structure*. The starting RMSD of the substrate, calculated based on comparison of both structures, was linearly decreased to 0 Å within a restrain period and then kept at 0 Å during the rest of the TMD simulation. Three different sets of input parameters were tested, differing in the duration of the restrain periods and the total length of the TMD simulation (Table 1). To prevent rotation of the entire molecule, the center of mass and orientation of the protein was fixed. Coordinates were stored every 2 ps.

### Non-restrained MD simulations

Non-restrained MD simulations were employed to study the functional and structural roles of GRL: specifically, (i) to study in detail the GRL-substrate interaction in two selected moments along the substrate translocation trajectory and (ii) to address the role of GRL in tertiary and quaternary structure of the MPP.

All non-restrained MD simulations were carried out using the AMBER suite [29] with the *parm99SB* force field [30]. The simulation protocol used was as follows. First, the protonation states of all histidine residues were set to create an optimal H-bond network. Next, all remaining hydrogen atoms were added using the *Leap* program from the AMBER package. The structures were charge-neutralized by adding an appropriate number or Na^+^ ions. To prevent rotation of the entire molecule, the center of mass and orientation of the protein were fixed. All systems were inserted in a rectangular water box filled by TIP3P water molecules; the layer of the water molecules was 9 Å thick. Each system was then minimized prior to the production phase of the MD run in the following way. The protein was frozen and the solvent molecules and counterions were allowed to move during a 1000-step minimization process followed by a 10-ps-long MD run under NpT conditions (i.e. p=1 atm, T=298.15 K). The side chains were then relaxed by several sequential minimizations with decreasing force constants applied to the backbone atoms. After relaxation, the system was heated to 50 K for 20 ps and then up to 298.15 K for 90 ps. The particle-mesh Ewald method for treating electrostatic interaction was used. For the production phase, all simulations were run under periodic boundary conditions in the NpT ensemble at 298.15 K and at a constant pressure of 1 atm using a 2-fs time integration step. The SHAKE algorithm with a tolerance of 10^−5^ Å was applied to fix all bonds containing hydrogen atoms. A 9.0 Å cutoff was used to treat non-bonding interactions. Coordinates were stored every 10 ps. The total durations of the production phases, the total number residues, atoms, counterions and water molecules of the studied systems are summarized in Table 3.

**Table 3:** Durations of production phases, and the number of amino acid residues, atoms and water molecules in the systems studied.

	**Total**	**Residues in**			**Ions**		**Water**	**Atoms in**
	**duration**	**α-MPP**	**β-MPP**	**substrate**	**Zn^2+^**	**Na^+^**	**molecules**	**total**
**Substrate in the MPP active site**								
WT MPP	100 ns	457	439	0	1	6	21547	78507
WT MPP + peptide	100 ns	457	439	11	1	3	21505	78576
ΔGRL MPP	100 ns	441	439	0	1	7	22311	80614
ΔGRL MPP + peptide	100 ns	441	439	11	1	4	22239	80590
**GRL-substrate interaction**								
after 0.48 ns of TMD	100 ns	457	439	12	1	3	21834	79563
after 0.84 ns of TMD	100 ns	457	439	12	1	3	21749	79308

### Data analysis

Several indicators were chosen for monitoring trajectory stability and conformational changes, including analyses of RMSDs, radius of gyration, secondary structure elements and the evaluation of inter-residue distances.

With regard to RMSD, three interpretations were used – time-based, residue-based and 2D RMSD analysis. In the case of time-based analysis, RMSDs were calculated in 0.1 ns intervals during the whole production part of MD simulation, as a measure of difference between the starting and present structures. RMSDs were also calculated for every residue between the starting structure and the structure obtained at the end of the MD simulation. All RMSDs were calculated using only the backbone Cα atoms of MPP, those of the substrate were not monitored. The MDTRA software package was used for all RMSD calculations [31]. The radius of gyration was calculated using AMBER [29] and the buried area per monomer upon dimerization was analyzed using the PDBePISA Server [32]. All figures showing protein structures were generated by PyMOL [33].

## Supporting Information

Figure S1 **Time-based and residue-based RMSD plots of WT MPP during a TMD simulation of substrate translocation from GRL to MPP active site.** A) The RMSD of backbone Cα atoms of WT MPP during a 1.8 ns TMD simulation. The red vertical lines mark the one-third (0.48 ns) and half-way (0.84 ns) points of the trajectory. The structures of these two steps were studied in detail using non-restrained MD simulations. B) The residue-based RMSD of WT MPP at the beginning and the end of the targeted MD simulation. Yellow, orange and green bars along the *x*-axes indicate the residues corresponding to the α- and β-MPP subunits and to the GRL, respectively. For comparison, in both graphs were used the same scale as those of Figures 4 and 5.
(TIF)

Figure S2 **2D plots of backbone Cα RMSDs during non-restrained MD simulations of WT and ΔGRL MPP.** Panels A and B show 2D plots of the change in the RMSD of the Cα atoms of the WT MPP structure over the course of a 100 ns simulation with respect to the initial model. Panel A shows WT MPP without a bound substrate and panel B shows the active site-bound form. Panels C and D show the same information for the free (C) and bound (D) forms of ΔGRL MPP.
(TIF)

Figure S3 **Radius of gyration of WT MPP and ΔGRL MPP without and with bound substrate in MPP active site.** Panel A shows the radius of gyration of the WT MPP structure over the course of the 100 ns simulation both with (red line) and without (blue line) a bound peptide substrate. Panel B shows the same information for the ΔGRL MPP structure. Note that for the WT structure, the substrate causes the radius to shrink while it increases greatly for the ΔGRL MPP structure.
(TIF)

Figure S4 **Alignments of the GRL regions of MPP and MPP-like proteins from selected organisms.** The region containing residues 285-300 of the α-MPP subunit that is missing in the ΔGRL MPP models is underlined.
(TIF)

Table S1 **Interaction surfaces between the α and β-subunits of MPP during TMD simulation.**
(DOCX)

Video S1 **Targeted MD simulation of the substrate translocation from the place of its recognition (i.e. from the GRL) to the site of its processing (i.e. to the MPP active site).** The restrain period is 1.6 ns and the total simulation time is 1.8 ns. α- and β-MPP subunits are displayed in yellow and orange color, respectively. The backbone trace of GRL is displayed as a red tube (residues 285-300) and K296, M298, Y299 and Y303 residues are shown in stick format. Peptide substrate is displayed as a tube in magenta color and R4, R8 (i.e. the R-2 motif) and F10 are shown in stick format. Zinc-binding residues H70, E73, H74 and E150 and the R-2-binding motif residues E160 and D164 of the β-MPP subunit are displayed as sticks. Zinc ion is shown as a blue sphere. Note that (i) the residues 289-393 of the GRL (green tube) are highly flexible and undergo major conformation changes during the whole substrate translocation process and that (ii) the R8 residue of the substrate is at the end of the simulation reoriented towards the E160 residue of the R-2-binding motif of the β-MPP subunit, just prior the subsequent proteolysis.
(ZIP)
