# Supplementary figures and images for: A Computational Study of the Glycine-Rich Loop of Mitochondrial Processing Peptidase
Source: PLoS One. 2013 Sep 13;8(9):e74518. doi: 10.1371/journal.pone.0074518 (PMC3772902; doi:10.1371/journal.pone.0074518)

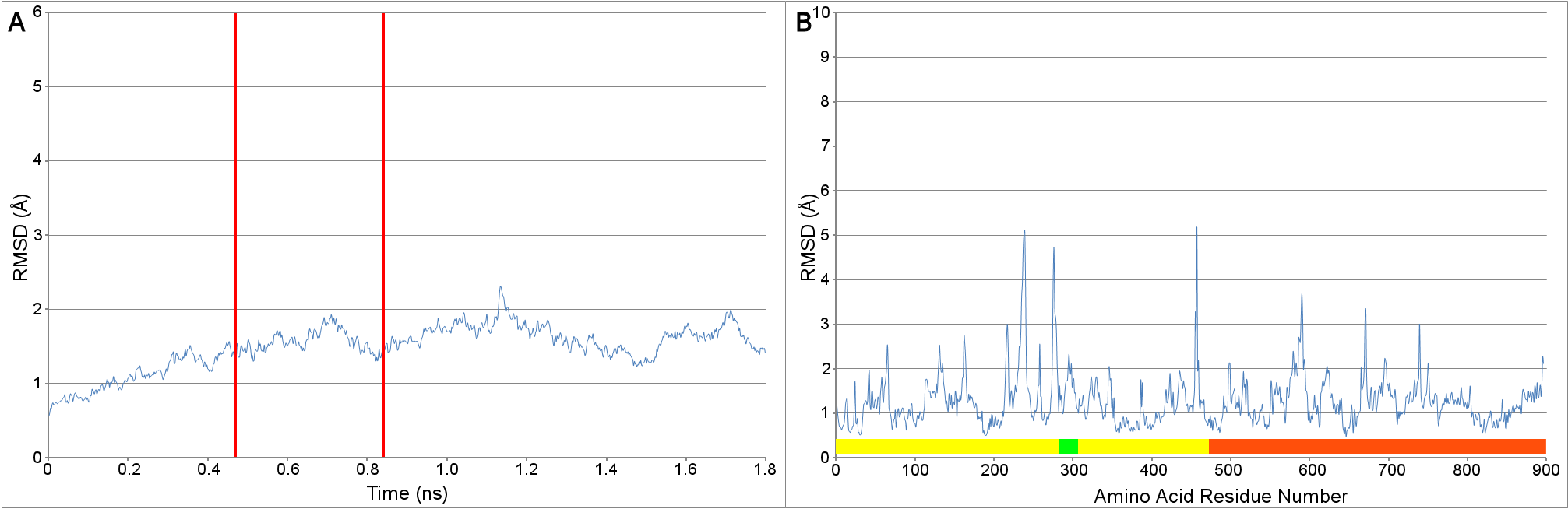

Supplement: Figure S1 — Time-based and residue-based RMSD plots of WT MPP during a TMD simulation of substrate translocation from GRL to MPP active site. A) The RMSD of backbone Cα atoms of WT MPP during a 1.8 ns TMD simulation. The red vertical lines mark the one-third (0.48 ns) and half-way (0.84 ns) points of the trajectory. The structures of these two steps were studied in detail using non-restrained MD simulations. B) The residue-based RMSD of WT MPP at the beginning and the end of the targeted MD simulation. Yellow, orange and green bars along the x-axes indicate the residues corresponding to the α- and β-MPP subunits and to the GRL, respectively. For comparison, in both graphs were used the same scale as those of Figures 4 and 5. (TIF) [file pone.0074518.s001.tif]

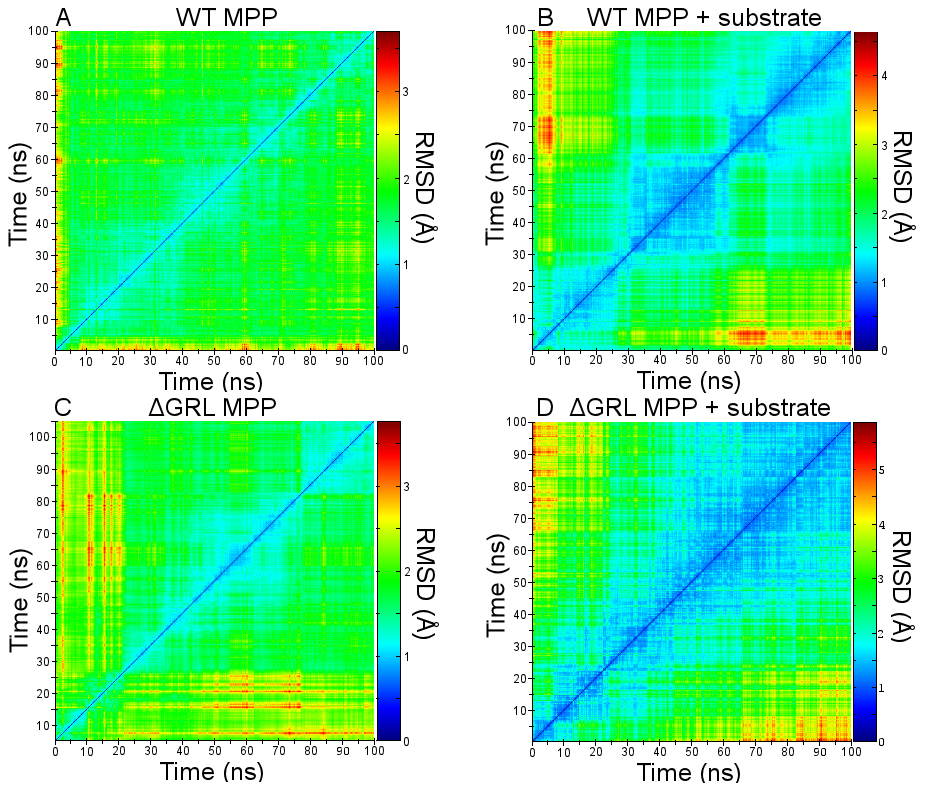

Supplement: Figure S2 — 2D plots of backbone Cα RMSDs during non-restrained MD simulations of WT and ΔGRL MPP. Panels A and B show 2D plots of the change in the RMSD of the Cα atoms of the WT MPP structure over the course of a 100 ns simulation with respect to the initial model. Panel A shows WT MPP without a bound substrate and panel B shows the active site-bound form. Panels C and D show the same information for the free (C) and bound (D) forms of ΔGRL MPP. (TIF) [file pone.0074518.s002.tif]

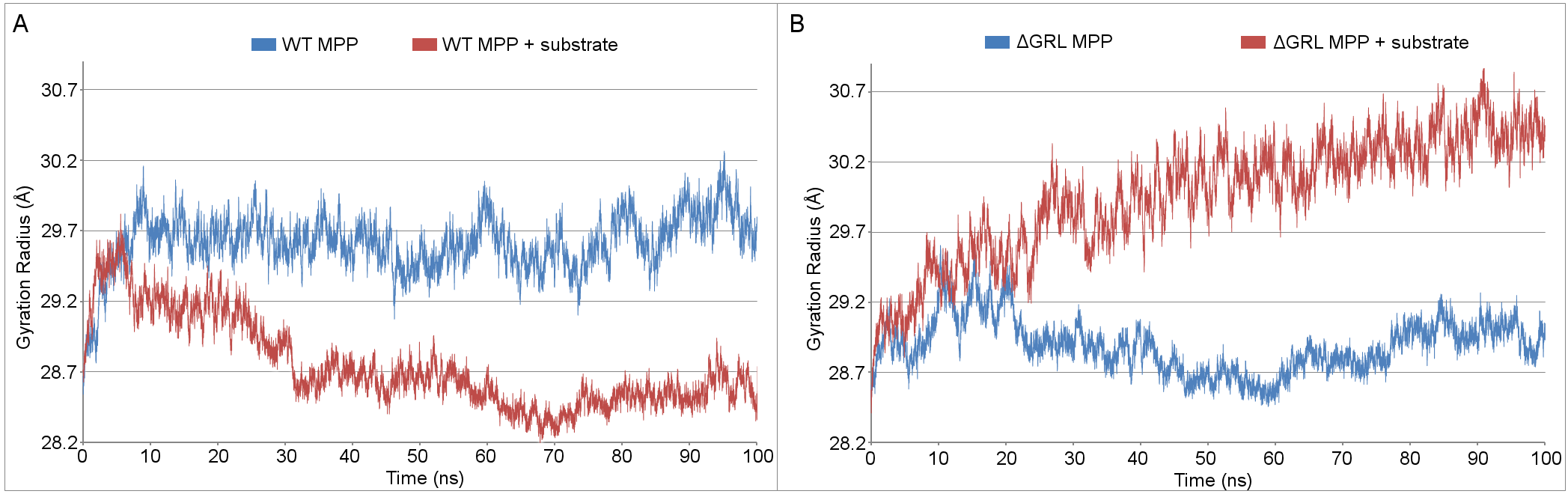

Supplement: Figure S3 — Radius of gyration of WT MPP and ΔGRL MPP without and with bound substrate in MPP active site. Panel A shows the radius of gyration of the WT MPP structure over the course of the 100 ns simulation both with (red line) and without (blue line) a bound peptide substrate. Panel B shows the same information for the ΔGRL MPP structure. Note that for the WT structure, the substrate causes the radius to shrink while it increases greatly for the ΔGRL MPP structure. (TIF) [file pone.0074518.s003.tif]

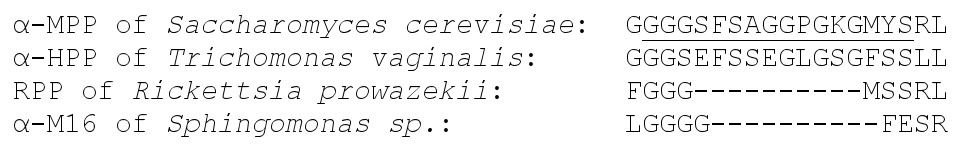

Supplement: Figure S4 — Alignments of the GRL regions of MPP and MPP-like proteins from selected organisms. The region containing residues 285-300 of the α-MPP subunit that is missing in the ΔGRL MPP models is underlined. (TIF) [file pone.0074518.s004.tif]
