# Supplementary material for: A Computational Study of the Glycine-Rich Loop of Mitochondrial Processing Peptidase
Source: PLoS One. 2013 Sep 13;8(9):e74518. doi: 10.1371/journal.pone.0074518 (PMC3772902; doi:10.1371/journal.pone.0074518)
Supplement: Table S1 — Interaction surfaces between the α and β-subunits of MPP during TMD simulation. (DOCX) [file pone.0074518.s005.docx]

| **Time** | **α-MPP versus β-MPP** |
| --- | --- |
| 0 ns | 2250 Å^2^ |
| 0.3 ns | 2290 Å^2^ |
| 0.6 ns | 2150 Å^2^ |
| 0.9 ns | 2200 Å^2^ |
| 1.2 ns | 2110 Å^2^ |
| 1.5 ns | 2120 Å^2^ |
| 1.8 ns | 2200 Å^2^ |
